# Reports of Two Cases of Cerebro-Spinal Menengitis

**Published:** 1867-03

**Authors:** Wm. O’Daniel

**Affiliations:** Marion, Ga.


*Report of two cases of Cerebro-Spinal Menengitis.* By Wm. O'Daniel, M. D., of Marion, Ga.

On the 19th of October last, at 2 o'clock, P. M., I was called to see Mrs. J——, 65 years of age, of feeble constitution, and stroumous diathesis. Upon my arrival, I was informed by her daughter, a very intelligent lady, that her mother was suffering intensely with sick headache—a disease that had caused her much suffering for a number of years.

Upon examination, I found her delirious, face flushed, eyes red, pupils much dilated, tongue furred, bowels constipated, pulse 110 and full, respiration hurried, and perspiring freely. She apparently suffered the most excruciating pain in the head. So great was her distress that she had to be held on the bed.

Twenty minutes after my arrival she had a convulsion, which lasted, perhaps, fifteen minutes: in half an hour she had another; and thus they continued to return for six hours, after which time the interval was one hour instead ot half an hour.

I ordered back of patient's head shaved, and a blister applied from the occiput to the first dorsal vertebræ, and frictions with oil turpentine the whole length of the spine, not covered with blister. Ordered the following: ℞ Hyd. chlor. mit. grs. xv., pulv. doveri, grs. x., m. ft. chart 3, and to be taken every two hours, followed in three hours after last dose by ol. recini ℥ ss., which evacuated the bowels by one o'clock. After the free evacuation of the bowels, I gave the following: ℞ Sulph. quinia grs. xxv., pulv. doveri grs. x., m. ft. chart 4., one to be taken every three hours, commencing at midnight. At this time I asked that Dr. D., an intelligent physician be called in consultation. Upon consultation, we were both convinced that the case was hopeless.

Called next morning, and found the patient irrational, pulse 98, small and tense. Convulsions ceased. Continued the sulphate quinine and dovers powder, and ordered hot mustard bath to feet and legs, and cold water to the head, every hour for three hours, by means of coffee pot held several feet from the head. Wine *pro re nata.*

Called in the evening, at 7 o'clock, and found patient resting well, respiration deep and laborious. Died next morning, at 6 o'clock, in a convulsion.

Case 2.—On the 14th of October last, at 7 o'clock, I was called to see John J——, an athletic black man, 22 years old, who, from childhood, had enjoyed good health. Upon my arrival, I was informed that two hours before, while in the yard, he was taken with a chill, and was unable to get back into the house without assistance. I found him perfectly delirious, pulse 100 and full; pupils much dilated. Ordered blister to back of neck, and gave him ℞ Calomel grs. xv., dovers powder grs. x., m. and make three powders, one to be taken every two hours; to be followed by a large dose castor oil. At midnight, he was to take the following: ℞ Sulph. quinia grs. xv., pulv. doveri grs. x., m. make three powders; one every three hours.

Called next morning at ten o'clock. Bowels had been evacuated. Found patient in semi-comatose condition; pulse 90 and full. Continue the quinine and dovers powders through the day, and ordered ten grains of dovers at ten o'clock P. M.

Called next morning at 7 o'clock, and found the patient much better: pulse 85, rational, and resting well. Ordered quinine, grs. xv., make three powders; one to be taken every three hours. Dovers powder grs. x., at ten o'clock, P. M. Wine *pro re nata.*

Called next morning at 7 o'clock, and found him improving rapidly: pulse 80 and almost natural. The patient was able to sit up in bed. The same treatment as the day before.

Called next morning at 7 o'clock. Found patient able to sit up: pulse normal. Prescribed wine *pro re nata*, and dismissed the case.

The patient rapidly recovered and in five days was able to labor, in the farm.

